# Identification of Mandarin Tones in Loud Speech for Native Speakers and Second Language Learners

**DOI:** 10.3390/bs15081062

**Published:** 2025-08-05

**Authors:** Hui Zhang, Xinwei Chang, Weitong Liu, Yilun Zhang, Na Wang

**Affiliations:** 1 School of International Education, Shandong University, Jinan 250100, China; hz.huizhang@sdu.edu.cn (H.Z.); 202100201015@mail.sdu.edu.cn (Y.Z.); 202202360032@mail.sdu.edu.cn (N.W.); 2 School of Education, University of Glasgow, Glasgow G12 8QQ, UK; 2977876c@student.gla.ac.uk; 3 Institute for Advanced Studies in Education, Shandong University, Jinan 250100, China

**Keywords:** Mandarin tone perception, categorical perception, loud speech, second language (L2) learners

## Abstract

Teachers often raise their vocal volume to improve intelligibility or capture students’ attention. While this practice is common in second language (L2) teaching, its effects on tone perception remain understudied. To fill this gap, this study explores the effects of loud speech on Mandarin tone perception for L2 learners. Twenty-two native Mandarin speakers and twenty-two Thai L2 learners were tested on their perceptual accuracy and reaction time in identifying Mandarin tones in loud and normal modes. Results revealed a significant between-group difference: native speakers consistently demonstrated a ceiling effect across all tones, while L2 learners exhibited lower accuracy, particularly for Tone 3, the falling-rising tone. The loud speech had different impacts on the two groups. For native speakers, tone perception accuracy remained stable across different speech modes. In contrast, for L2 learners, loud speech significantly reduced the accuracy of Tone 3 identification and increased confusion between Tones 2 and 3. Reaction times in milliseconds were prolonged for all tones in loud speech for both groups. When subtracting the length of the tones, the delay of RT was evident only for Tones 3 and 4. Therefore, raising the speaking volume negatively affects the Mandarin tone perception of L2 learners, especially in distinguishing Tone 2 and Tone 3. Our findings have implications for both theories of L2 tone perception and pedagogical practices.

## 1. Introduction

Speakers often modify their speech patterns to enhance intelligibility, adjusting to the listeners’ characteristics or specific communication contexts, such as infant-directed speech (IDS), foreigner-directed speech, speech in noise (Lombard speech), and speech with increased talker-to-listener distance (loud speech) (Cox et al., 2023; Marklund & Gustavsson, 2020; Piazza et al., 2022; C. Shen et al., 2023; Shih & Lu, 2015). In second language (L2) teaching, for instance, teachers frequently raise their vocal volume in the classroom to increase intelligibility or capture students’ attention (Mota et al., 2021; Rantala et al., 2015; Uther et al., 2007). Lindblom’s (1990) H & H model suggests that speech articulation ranges along a scale from highly distinct (hyperarticulated) to more casual and reduced (hypoarticulated). For instance, in challenging auditory settings like noisy backgrounds, speakers are inclined to produce clearer, more exaggerated speech, whereas in favorable conditions, they tend to speak more effortlessly and with reduced precision (Lindblom, 1983). This variation stems from a speaker’s adjustments based on communicative demands, aiming to ensure listener comprehension while minimizing articulatory effort. The H & H theory mostly analyzes the dynamic adjustments of speech from the speakers’ perspectives, while offering limited discussion on whether the hyper-articulation aims for a clearer and easier perception of speech categories, i.e., the perceptual significance. Therefore, exploring how listeners perceive loud speech offers insights into the perceptual significance of speech adjustments, especially for L2 learners who are not as proficient as adult-native listeners, adds theoretical implications on L2 speech learning, and provides practical recommendations for L2 classroom teaching.

### 1.1. Acoustic Modifications in Loud Speech

A bulk of documents have demonstrated that increased speech loudness is associated with a series of physiological changes, including increased lung volume, higher subglottal pressure, higher rate of vocal fold vibration, and greater articulatory displacement (Dromey & Ramig, 1998; Huber & Chandrasekaran, 2006; Garnier et al., 2018; Koenig & Fuchs, 2019; Ladefoged & McKinney, 1963; Myers & Finnegan, 2015; Schulman, 1989). In the suprasglottal part, loud speech often widens tongue constriction and jaw opening and lowers tongue height (Carding & Mathieson, 2018; Fant, 1971; Huber & Chandrasekaran, 2006; Mefferd, 2017; Schulman, 1989). These physiological modifications are accompanied by acoustic changes in multiple dimensions, such as a longer syllable duration, an increase in sound intensity, a higher mean fundamental frequency (F0), expended F0 range (Belz et al., 2023; Neel, 2009; Pagel et al., 2024; Picheny et al., 1986; Schulman, 1989; Lunichkin et al., 2023; Wilson Black et al., 2024; Zhao & Jurafsky, 2009), and a series of formant changes (Dromey et al., 1995; Ternström et al., 2006).

Speech modes that have stronger loudness influence the acoustic characteristics of lexical tones. In Lombard speech, all Cantonese tones^1^ experienced a universal increase in intensity and F0 when produced in noisy environments. However, the modification of F0 did not result in significant expansion or increased dispersion of the tone space, indicating that the primary adjustment in Lombard speech was an overall enhancement of phonetic strength rather than sharper tonal contrast (Zhao & Jurafsky, 2009).

Shih and Lu (2015) investigated the acoustic modifications of Mandarin tones with stronger loudness due to increased talker-to-listener distance (TLD). In Mandarin, there are four lexical tones responsible for discriminating lexical meanings. The four tones are categorically distinguished by their respective patterns of F0 variation: Tone 1, a high-level tone (classified as 55 in the traditional tonal number system); Tone 2, a mid-rising tone (35); Tone 3, a mid-falling-rising tone (214); and Tone 4, a high-falling tone (53) (Blicher et al., 1990; Chao, 1965; Howie, 1976). Tone 1 maintains a consistently high F0 throughout the syllable. Tone 2 starts with a relatively low F0 and rises in the remainder of a syllable. Tone 3 has a dipping contour, which is a curve opening upward. Tone 4 starts with the highest F0 among the four tones, then drops dramatically until the end of a syllable (Xu, 1997).

The evident changes were the stronger intensity, higher F0, and longer syllables, which were caused by more vocal efforts. Furthermore, loud speech led to greater acoustic distinction between Tone 3 and other tones concerning the maximal F0 (Shih & Lu, 2015). However, slope and curvature^2^ were not found to change along with increasing TLD. Although the authors did not explicitly compare the distinction among tones in loud speech and normal speech concerning duration and intensity, the model summary outputs showing the interaction of levels of distance and tones indirectly proved that duration was not differentiated more in loud speech.

Similar to Shih and Lu (2015), Zhang et al. (2023) elicited loud speech with a listener standing five meters away outside a sound-attenuated booth and examined the tonal modifications. Loud speech resulted in stronger intensity, increased F0, and longer duration, consistent with Shih and Lu (2015). Furthermore, Zhang et al. (2023) also found expanded tonal space (i.e., greater separation among tones) by showing that the critical acoustic cues for discrimination of a tone pair (e.g., height for Tone 1 and Tone 3, slope for Tone 1 and 4, Tone 1 and 2, and curvature for Tone 2 and 3) were more differentiated in loud speech than in normal speech. Similar to Shih and Lu (2015), duration and intensity did not result in a larger dispersion of tonal space.

### 1.2. Perceptual Correlates of the Acoustic Modifications in Loud Speech

Understanding how the F0 modifications in loud speech influence tone perception among native and L2 learners is essential, as the acoustic differences between normal and loud speech directly impact key cues in Mandarin tone perception (Oxenham, 2012; Tong et al., 2014).

The dynamic characteristics of F0 contours play a critical role in distinguishing these tones (Leung & Wang, 2020; Tupper et al., 2020). These dynamic features are reflected in various dimensions of F0, such as F0 height, slope, curvature, as well as onset and offset values. F0 height is a particularly significant cue for distinguishing Tone 1 from Tone 3 (Tsao, 2017). F0 slope helps distinguishing Tone 1 from Tone 4, Tone 1 from Tone 2, Tone 2 and Tone 4, and Tone 3 and Tone 4 (F. Chen & Peng, 2016; Gandour, 1983; Hallé et al., 2004; Huang & Holt, 2009; B. Li & Zhang, 2010; Zhang et al., 2022). Additionally, the curvature is crucial for distinguishing between Tone 2 and Tone 3, as it captures details that define these tones (Shih & Lu, 2015; Tupper et al., 2018). The location of the F0 turning point, i.e., where the trajectory of F0 across time starts to change direction, provides an important cue for Tone 2 and Tone 3 discrimination (X. S. Shen & Lin, 1991; Wu & Lin, 2008). For example, Tone 2 has a less pronounced initial F0 fall than Tone 3 and reaches its turning point earlier. Manipulating this turning point can significantly affect the perception of Tone 2 and Tone 3 (X. S. Shen et al., 1993). The F0 onset and offset are also crucial acoustic cues for Mandarin tone perception (Smith & Burnham, 2012). Listeners were able to accurately identify Mandarin tones even when only the initial and final segments of the tones were presented, suggesting that the removal of the central portion did not significantly impair identification accuracy among native speakers (Gottfried & Suiter, 1997; Lee et al., 2009).

Duration and amplitude play conditional roles in tonal perception. The role of amplitude and duration in tone perception becomes evident when F0 information is missing in whispered speech (Whalen & Xu, 1992; Zhang et al., 2022; Liu & Samuel, 2004). Among the above-mentioned cues important for tone perception, many demonstrate modifications in loud speech. However, it remains unclear how the perceptual influence of the acoustic modifications in loud speech affects the perceptual behavior of native listeners and L2 learners.

### 1.3. Tone Perception for Native and L2 Learners

Tone identification of native listeners usually reaches a ceiling effect in quiet environments with resistance to moderately adverse communicative situations, such as noise, incomplete acoustic signals, and variations (Gottfried & Suiter, 1997; Lee et al., 2009; M. Li et al., 2016; Shih & Lu, 2015). The performance of tone perception among L2 learners, who are often reported to struggle with tone perception, in contrast, fluctuates easily with the subtle acoustic modifications in various communicative situations. Foreigner-directed speech (FDS) facilitates L2 perception and learning by employing features such as vowel hyperarticulation, slower speech rates, and increased pauses (S. Figueiredo, 2019; Y. Zhu & Mok, 2022). These adjustments provide clearer speech signals, making it easier for non-native listeners to process and understand. Additionally, variations in intensity and pitch emphasize critical linguistic cues, with adaptive modifications based on listener feedback enhancing communication efficiency (Piazza et al., 2022).

At the same time, the perception of tones by L2 learners is also highly susceptible to adverse acoustic conditions. For instance, X. Chen et al. (2020) found that background noise significantly impaired the ability of L2 learners to perceive Mandarin tones. Similarly, multi-talker babble, particularly involving speakers of the same language or familiar accents, disrupted the recognition of target words (Van Engen & Bradlow, 2007; Lee et al., 2009; Leung & Wang, 2020). These findings highlight the complexity of tone perception, as specific acoustic characteristics in communicative contexts can either enhance or inhibit performance (S. Figueiredo, 2024; S. A. D. B. Figueiredo & Silva, 2009; Uchihara et al., 2024). Clear speech with enhanced acoustic features tends to improve perception, while degraded signals reduce it. Among the Mandarin tones, difficult and confusable tones (such as Tone 2 and Tone 3) are particularly vulnerable to changes in the acoustic environment, whereas easily intelligible tones (such as Tone 1 and Tone 4) remain relatively stable across different conditions (Chow et al., 2019; J. I. Han & Tsukada, 2020; Hao, 2018; G. Shen & Froud, 2016). For example, since Mandarin Tone 2 and Tone 3 have similar F0 contours, the perception of these two tones can easily be confused in the presence of noise and babbling (Mao & Xu, 2017; Shi, 2019). In contrast, Mandarin Tone 4 has distinct acoustic characteristics: a high falling pitch and a short duration, so even hearing-impaired children perform better in identifying this tone in noise (S. C. Peng et al., 2004; S. Zhu et al., 2014).

The difficulty with Tone 2 and Tone 3 arises from their similar contour shapes, which differ only subtly in ways that L2 learners have not yet developed the perceptual sensitivity to distinguish. The problem of Tone 2–3 confusion is even independent of L2 learners’ tonal experience in their mother languages (Hao, 2012; So & Best, 2010). For example, even with tonal experiences, Cantonese Mandarin-naïve listeners faced more challenges with Mandarin Tone 2 and Tone 3 discrimination than Mandarin-naïve listeners without any tonal experiences (English and Japanese). The possible reason is that Mandarin Tone 2 and Tone 3 are both similar to one Cantonese tonal category, i.e., Tone 2. In such cases, the experiences in the L2 learners’ native tonal inventory sometimes form an obstacle instead of facilitation.

Even for native Thai speakers learning Mandarin as L2, who are familiar with both high-rising and falling-rising tones in their L1 (Wu et al., 2014; J. Chen et al., 2023), the assimilation of Mandarin Tone 3 to tones in their L1 remains inconsistent after 0.5–2 years of learning. Thai, like Mandarin, is a tone language, but the two systems differ in both the number and acoustic characteristics of tonal categories. Thai includes five tones—typically described as mid-level, low-falling, high-falling, rising, and falling-rising—compared to the four Mandarin tones introduced above. While there are points of acoustic resemblance, especially between Mandarin Tone 3 and the falling-rising Thai tone, the mapping is not absolute. For instance, in connected Mandarin speech, Tone 3 is frequently realized as a low-falling contour, which may phonetically resemble Thai’s low-falling tone. Most Thai L2 learners highlighted pitch height and deemed Mandarin Tone 3 as being similar to the low-falling tone in their mother language, while other learners emphasized the dipping contour and considered the nature of Mandarin Tone 3 as being similar to the falling-rising tone in their mother language (Wu et al., 2014). The former erroneously rejected Tone 3 identification for tone productions that involve obvious rising or high pitch. The understanding of Tone 3 as being a low-falling tone, even for monosyllables, does not change as experiences with Mandarin accrue (Wu et al., 2014). Therefore, predicting tonal challenges for L2 learners whose native languages are tonal is difficult due to the complex relationship between the tonal systems of Mandarin and their first languages. Negative transfer from the mother language could result in an inaccurate understanding of the key feature of Mandarin tones, thus bringing about perceptual error, especially when the situation is even worse with acoustic variability, such as elevated F0 in loud speech in the present study.

In Shih and Lu (2015), speech stimuli with normal and increased loudness were presented to native speakers and elementary L2 learners. The results showed that native listeners’ perception was unaffected by the acoustic modifications in loud speech, while L2 learners showed improved identification of Tone 3 under loud conditions. However, it remains unclear whether this improvement was due to increased intensity alone, as the intensity levels of normal and loud speech were not normalized. In other words, it is possible that the normal speech did not reach the auditory threshold necessary for accurate tone recognition among L2 learners, and that the increased intensity associated with larger TLD simply made the tones more audible. If so, the observed improvement may be attributed to louder volume rather than to acoustic modifications intended to enhance tonal contrast. Therefore, further research is needed to determine whether L2 learners benefit from specific tonal enhancements beyond a general increase in intensity.

The impact of F0 modifications in loud speech on L2 tone perception is complex and potentially contradictory. On the one hand, these F0 modifications that enhanced the distinction among tones in loud speech are assumed to improve L2 learners’ ability for tone perception, particularly for learners with an underdeveloped tonal inventory. Nevertheless, the concomitant rising of pitch height complicates the issue, as pitch height is critical for distinguishing Tone 2 from Tone 3, and Tone 3 is primarily characterized by its low pitch, especially for L2 learners who rely on pitch height to discriminate Tone 2 and Tone 3. A rise in pitch height may cause L2 learners to misidentify Tone 3 as Tone 2. This introduces a paradox: while some acoustic features of loud speech may improve tone perception accuracy, the rise in pitch height may have the opposite effect. Consequently, it remains unclear which aspect exerts a greater influence, and this study seeks to address this question.

The present study is significant because it seeks to disentangle the role of stronger intensity in loud speech from the acoustic modifications that are thought to enhance tonal contrast, by controlling intensity. This research contributes to a more precise understanding of the perceptual significance of the speakers’ strengthened vocal efforts in loud speech. Specifically, it clarifies whether, except for increasing volume, the speakers also attempt to make tones clearly distinguished, and the L2 learners really benefited from the speakers’ efforts. Furthermore, the present study adds more data to the perceptual significance of various speech modes from the perspective of L2 learners, providing theoretical insights into the nature of L2 perception and pedagogical implications for the design of effective pronunciation training strategies in tonal language classrooms.

### 1.4. The Present Study

This study builds on previous research on how speech modifications influence tone perception by examining the impact of normal and loud speech on the tone perception of adult native listeners and L2 learners. The primary goal is to determine whether the acoustic changes associated with loud speech improve tone perception in L2 learners. To address this, we selected 22 adult Thai L2 learners and 22 native speakers of Mandarin to assess their tone perception performance under both normal and loud speech conditions. If listeners’ accuracy improves in loud speech, it suggests that the acoustic modifications associated with loud speech enhance tone perception. If no significant change in accuracy is observed, it implies that different speech modes have little effect on tone perception. Otherwise, if accuracy decreases, this will indicate that loud speech has a negative impact on the tone perception of L2 learners. This study will therefore elucidate the nuanced effects of speech modes on tone perception, providing valuable insights for Mandarin L2 teaching.

## 2. Materials and Methods

### 2.1. Subject

This study involved a total of 44 participants, comprising 22 native Chinese speakers and 22 Thai learners of Chinese. All native speakers (11 males and 11 females) were sophomores of Shandong University, aged between 18 and 20 years, using Mandarin as their primary language alongside occasional dialects in daily life. The Thai learners (7 males and 15 females), aged between 18 and 25 years, were students at universities in mainland China. Mandarin learning experience of L2 learners ranged from 7 months to over 14 years, with varying HSK levels: 4 learners below HSK-4, 6 learners at HSK-4, 9 learners at HSK-5, and 3 learners at HSK-6. The eligibility criteria for the sample required that participants report no hearing, language, or speech difficulties and that they had not received any regular musical training in the past five years. After the experiment, they all received 75 RMB as compensation.

### 2.2. Stimuli

The stimuli for the perception experiment were obtained from recordings made by native Mandarin speakers. Twenty commonly used Chinese characters were selected, representing five syllables ([pa], [ti], [kʰɤ], [wu], [y]) and four tones: high-level tone (Tone 1), rising tone (Tone 2), falling-rising tone (Tone 3), and falling tone (Tone 4). These characters were read by 30 undergraduates at Shandong University (15 males and 15 females) in a sound-attenuated booth via Praat (rate: 44,100 Hz). Each speaker read the characters in normal speech mode followed by loud speech mode, with normal speech always preceding loud speech to prevent any carryover effect. Based on pronunciation clarity and accuracy, the researchers selected recordings from 20 speakers (10 males and 10 females) for the perceptual experiment. The recordings were then segmented into monosyllabic words, and the intensity of all stimuli was normalized to 79 dB to eliminate any potential impact of intensity level on perceptual accuracy.

### 2.3. Procedure

The experiment was executed via the online platform, Gorilla. Participants were instructed to perform the task in a quiet environment while wearing headphones. Before the experiment, participants joined a Tencent Meeting, where the investigators checked their environment and equipment by asking them to turn on both their microphones and cameras. Once this check was completed, participants were provided with the link to the Gorilla platform.

The experiment used a randomized block design. The procedure consisted of one questionnaire and one block of trials. The questionnaire gathered demographic information, such as the first language, any other language background, the length of Chinese learning, and any professional musical training experience. The block contained 10 practice trials followed by 796 experimental trials (396 loud speech and 400 normal speech), with participants being allowed to break every 80 trials. The practice stimuli were excluded from the test data. During each trial, a fixation point was displayed for 500 ms, followed by an audio stimulus. Four prompts (Press “1”, Press “2”, Press “3”, and Press “4”) were displayed on the screen, which respectively had a visual display of pinyin and tone information below. Participants responded based on the audio stimulus they heard, with no time limit for responses. The entire experiment lasted approximately 50 min.

All procedures involving human participants were conducted in accordance with the Helsinki Declaration. The ethical review and approval were waived in accordance with Article 32 of the Measures for Ethical Review of Life Sciences and Medical Research Involving Human Beings in China. All participants provided informed consent and were assured of the anonymity and confidentiality of their responses. They were also informed of their right to withdraw from the study at any time without any negative consequences. We have retained the information about informed consent and participants’ rights.

## 3. Analysis and Results

This section presents analyses of the effects of group (categorical factor: native listeners vs. L2 learners), tone (categorical factor: Tone 1, Tone 2, Tone 3, and Tone 4), and condition (categorical factor: loud condition vs. normal condition) on response accuracy and reaction time (RT). Specifically, the analysis addresses two main questions: (1) How do Thai L2 learners perform on each tone? (2) How does speech condition (normal vs. loud) affect tone perception accuracy and reaction time for both native listeners and L2 learners?

We built a generalized linear mixed-effects model (Response accuracy~Group × Tone × Mode + (1 + Group + Tone + Mode | Subject)) using the *glmer ()* function from the *lme4* package in R to examine the main effects and interactions of group, tone, and condition on perceptual accuracy. The main effects and interaction effects were obtained by using *anova* function. Post-hoc comparisons were conducted using the emmeans() package.

Similarly, a linear mixed-effects model (RT~Group × Tone × Mode + (1 + Group + Tone + Mode | Subject)) was built to analyze the effect of tone, group, and condition, as well as their interaction effects on RT. Ahead of model building, we removed raw RT data less than 200 ms and greater than 2000 ms and outliers greater than three standard deviations. The main effects, interaction effects, and post hoc comparisons were analyzed using the same method as perceptual accuracy.

In order to provide more information about how learners Chinese proficiency and gender influence the perceptual performance, we subset learners’ data and ran a generalized linear mixed-effects model (Response accuracy~Tone × Mode × HSKlevel + Gender + Gender:Tone + Gender:Mode + Gender:Tone:Mode + (1 + Tone + Mode | Subject)) and a linear mixed-effects model (RT~Tone × Mode × HSKlevel + Gender + Gender:Tone + Gender:Mode + Gender:Tone:Mode + (1 + Tone + Mode | Subject)).

### 3.1. Accuracy

**Figure 1:**
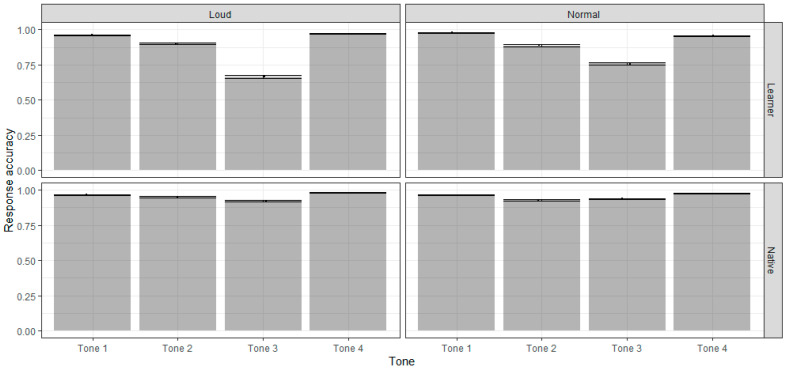
Response accuracy across four tones and two speech modes in native speakers and L2 learners.

Figure 1 presents the response accuracy of both native listeners and L2 learners across the four tones under normal and loud conditions. Native listeners consistently outperformed L2 learners for all tones under both speech conditions. For L2 learners, Tone 3 posed the most difficulty (76%), while their accuracy of Tone 1 and Tone 4 exhibited near-ceiling effects (Tone 1: 98%; Tone 4: 95%).

The generalized linear mixed-effects model (see Supplementary Materials for complete results) revealed a significant main effect of group (*F* (1, 43) = 33.05, *p* < 0.05), indicating that native speakers had generally higher accuracy in perceiving Mandarin tones than L2 learners. L2 learners demonstrated significantly lower accuracy than native speakers for Tone 3 (*ß* = −1.573, *SE* = 0.241, *p* < 0.001). However, there was no significant difference between the two groups for Tone 1, Tone 2, and Tone 4 (*ß* = 0.047, *SE* = 0.305, *p* = 1.00 for Tone 1, *ß* = −0.813, *SE =* 0.252, *p* = 0.091 and *ß* = −0.713, *SE* = 0.275, *p* = 0.404 for Tone 4). To quantify the proportion of variance explained by the model, we computed marginal and conditional R^2^ values. The marginal R^2^ was 0.18, indicating that the fixed effects accounted for 18% of the variance in response accuracy. The conditional R^2^ was 0.33, suggesting that the full model, including random effects for subjects, explained 33% of the variance.

**Figure 2:**
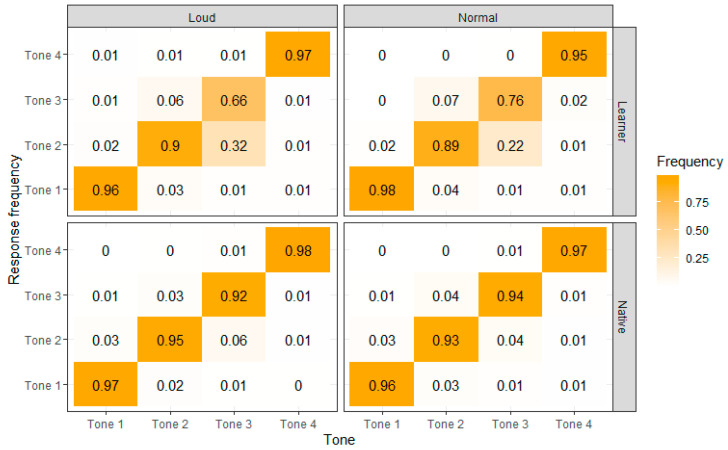
Response frequency across four tones and two speech modes in native speakers and L2 learners.

Simultaneously, a confusion matrix was constructed, as illustrated in Figure 2. This matrix displays both the distribution and percentage of tone choices made by listeners for each tone. The x-axis represents the correct responses, while the y-axis corresponds to the participants’ actual choices. The color gradient represents the percentage of responses, with darker shades indicating higher response rates and lighter shades indicating lower ones. As shown in Figure 2, the misidentification of both Tone 2 and Tone 3 for L2 learners derives from the confusion between Tone 2 and Tone 3. Specifically, the majority of misidentification of Tone 3 went to Tone 2 (32% out of 34% in loud condition and 22% out of 24% in normal condition). Similarly, most of the misidentification of Tone 2 went to Tone 3 (6% out of 10% in loud condition and 7% out of 11% in normal condition).

Based on the illustration of perceptual accuracy in Figure 1 and the confusion matrix in Figure 2, the performance of native listeners across the two speech modes remained generally unchanged (97% in loud speech and 96% in normal speech for Tone 1, 95% in loud speech and 93% in normal speech for Tone 2, 92% in loud speech and 94% in normal speech for Tone 3, and 98% in loud speech and 97% in normal speech for Tone 4). For learners, in contrast, the accuracy of Tone 3 perception for learners declined in the loud speech versus the normal speech, from 76 percent to 66 percent. The accuracy of perceiving the other three tones had no significant changes (96% in loud speech and 98% in normal speech for Tone 1, 90% in loud speech and 89% in normal speech for Tone 2, and 97% in loud speech and 95% in normal speech for Tone 4). Figure 2 shows that the dropped accuracy of Tone 3 in loud speech exactly corresponds to the rise of the misjudgment rate of Tone 2, suggesting that loud tones caused more Tone 3 to be misidentified as Tone 2.

The interaction effect for tone and condition was significant (*F* (3, 41) = 23.23, *p* < 0.001), but the tone × group × condition interaction effect was not statistically significant (*F* (3, 41) = 1.26, *p* > 0.05). The tone × condition interaction effect indicated that the effect of the condition was different across the four tones. Results of the post hoc comparisons showed that the performance of native listeners native listeners did not change across loud and normal conditions (Table 1). In contrast, the L2 learners’ accuracy dropped significantly when identifying Tone 1 and 3 in loud condition (*ß* = −0.678, *SE* = 0.184, *p* = 0.021 for Tone 1 and *ß* = −0.548, *SE* = 0.080, *p* < 0.001 for Tone 3), with the others remaining unchanged (*ß* = 0.137, *SE* = 0.105 *p* = 0.996 for Tone 2, and *ß* = 0.412, *SE* = 0.164, *p* = 0.486 for Tone 4).

The model (see Supplementary Materials for the complete outputs) involving Chinese proficiency and gender as fixed predictors showed that neither HSK level (*F* (1, 18236) = 0.689, *p* = 0.406) nor gender affected the perceptual accuracy (*F* (1, 18236) = 0.088, *p* = 0.767).

### 3.2. RT

**Figure 3:**
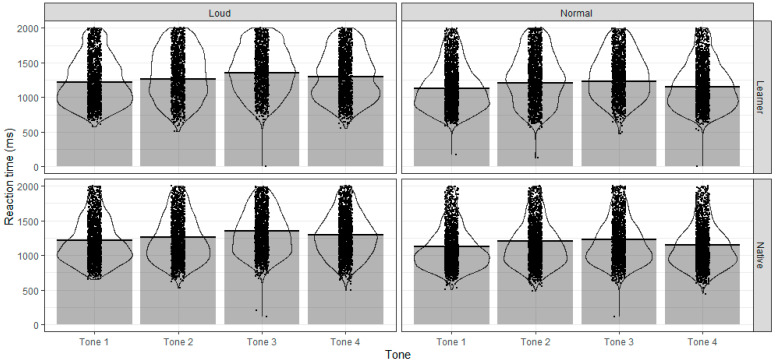
RT across four tones and two speech modes in native speakers and L2 learners.

Figure 3 shows the RT of listeners to four tones in normal and loud speech. Visually, there was no obvious difference between L2 learners and native listeners in RT. For both groups, responses of Tone 3 took the longest time to respond than the other three tones. Furthermore, RT in loud speech was longer than that in normal speech for both groups.

The linear mixed-effects model (see Supplementary Materials for complete results) showed that there was a significant effect of group on RT(*F* (1, 43) = 4.90, *p* = 0.044), though the difference between the two groups was subtle (56.71 ms). Post hoc comparisons confirmed that the difference in RT between learners and native listeners was not significant (Table 2). This suggested that L.2 learners generally react as quickly as native listeners in identifying tone, though they might give inaccurate responses. Further, results of the model showed that there was a significant interaction between tone and group (*F* (3, 41) = 50.70, *p* < 0.001) and a significant interaction effect between tone and condition (*F* (3, 41) = 25.40, *p* < 0.001), but the interaction effect of tone, group, and condition was not significant (*F* (3, 41) = 1.17, *p* = 0.318). The marginal R^2^ of the model was 0.07, and the conditional R^2^ was 0.43.

Post hoc comparisons showed that loud had the most effect on prolonging the RT of Tone 4 for both groups (*ß* = 148.57, *SE* = 16.3, *p* < 0.001 for native listeners, with a difference of 153 ms, and *ß* = 137.80, *SE* = 16.5, *p* < 0.001 for L2 learners, with a difference of 144 ms). Tone 3 was identified 140 ms slower in loud condition than normal condition for native listeners (*ß* = 139.65, *SE* = 16.5, *p* < 0.001) and 96 ms slower for L2 learners (*ß* = 101.92, *SE* = 18.1, *p* < 0.001). Compared with Tone 3 and Tone 4, the degree of extension of RT in loud condition was smaller for Tone 1 and Tone 2 (Tone 1: 95 ms longer identifying loud tones than normal tones for native listeners, *ß* = 93.99, *SE* = 16.3, *p* < 0.001 and 86 ms longer identifying loud tones than normal tones for L2 learners, *ß* = 91.54, *SE* = 16.4, *p* < 0.001; Tone 2: 74 ms longer identifying loud tones than normal tones for native listeners, *ß* = 75.90, *SE* = 16.4, *p* < 0.01 and 40 ms longer identifying loud tones than normal tones for L2 learners, *ß* = 59.37, *SE* = 17.1, *p* = 0.044), as Table 2 shows.

**Figure 4:**
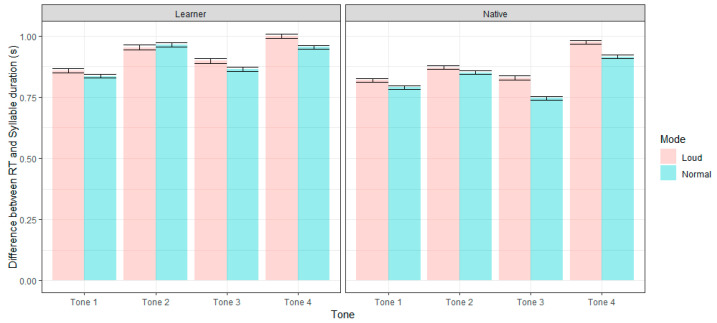
Difference between reaction time and tonal duration across four tones and two speech modes in native speakers and L2 learners.

In order to clarify whether the increase of RT was caused by perceptual difficulty or lengthened duration, we also calculated the difference between RT and tonal duration and then checked its variation across the two speech modes (Figure 4). The difference between RT and duration was submitted to a mixed linear effect model with mode, Tone, and group as fixed predictors (Difference~Mode × Tone × Group + (1 | Subject) + (1 | Syllable)). Post hoc comparisons showed that when considering the duration of loud tones, the lengthening of RT when identifying loud tones was significant for Tone 3 (*ß* = 0.081, *SE* = 0.009, *p* < 0.001 for native listeners, with a difference of 83 ms, and *ß* = 0.046, *SE* = 0.013, *p* = 0.024 for L2 learners, with a difference of 34 ms) and Tone 4 (*ß* = 0.057, *SE* = 0.009, *p* < 0.001 for native listeners, with a difference of 59 ms, and *ß* = 0.052, *SE* = 0.010, *p* < 0.001 for L2 learners, with a difference of 46 ms), which was consistent with the findings of total RT, as shown in Table 3. We also found significant lengthening of RT for learners’ Tone 1, but considering its marginal amount (only 22 ms), we deemed it as unchanged. Similarly, marginal and conditional R^2^ values of this model were computed, with 0.06 and 0.35, respectively. The model (see Supplementary Materials for the complete outputs) involving Chinese proficiency and gender as fixed predictors showed that neither HSK level (*F* (1, 19) = 0.005, *p* = 0.943), nor gender (*F* (1, 19) = 0.647, *p* = 0.431) affected the perceptual accuracy.

In summary, increased speech loudness impaired L2 learners’ perception of Tone 3 and intensified their confusion between Tone 2 and Tone 3. In contrast, native speakers’ tone identification remained unaffected by the acoustic changes in loud speech. Regarding reaction time, loud speech led to longer recognition times, particularly for Tone 3 and Tone 4.

## 4. Discussion

### 4.1. Challenges of Tone Perception for Thai L2 Learners

We examined the perceptual significance of Mandarin tones for native and Thai L2 learners from three aspects, i.e., response accuracy, confusion, and RT. Thai L2 learners exhibited an overall lower accuracy in distinguishing tones than native listeners, which indicates a notable struggle with distinguishing Mandarin tones, particularly Tone 2 and Tone 3^3^. This aligns with previous research indicating that L2 learners often struggle with tone recognition due to their native phonetic systems (Cao et al., 2024; X. Chen et al., 2020; Pelzl et al., 2021; G. Shen & Froud, 2019).

Analysis of the confusion matrix shows that the challenge with Tone 2 and Tone 3 discrimination derives from the misidentification of Tone 3 as Tone 2, which is also reported in the existing literature (Cao et al., 2024; Hao, 2018; X. Li et al., 2017). Results of the present study echo Wu et al. (2014), which found that most of the Thai native listeners assimilated monosyllabic Mandarin Tone 3 to Thai low falling. Consequently, Mandarin Tone 3 productions with a discernible final rising or a F0 height that is not at the bottom of a speaker’s pitch range tend to yield Tone 2 identification among Thai L2 learners. This often happens in loud speech since the low feature of loud Tone 3 is weakened. The pattern of confusion of Thai L2 learners is in line with that of learners from other language backgrounds. When identifying tone variants on the continuum from Tone 2 to Tone 3, Japanese L2 learners, who are from a pitch-accent language, had a narrower perceptual space for Tone 3 and thus a wider perceptual space for Tone 2 than native listeners, indicating that there were tones that the Japanese L2 learners labeled as Tone 2 but native listeners labeled as Tone 3 (Sun et al., 2018). The discrepancy between the perceptual decisions of learners and native listeners thus proved the misidentification of Tone 3 as Tone 2. A later study on English L2 learners, who are from an intonation language, showed a similar confusion pattern, with more responses of Tone 2 and less Tone 3 than native listeners for the tone variants along the Tone 2 and Tone 3 continuum (Jiang, 2023). The common problem of misidentification of Tone 3 as Tone 2 that exists among L2 learners, both with (Thai learners in the present study) and without (Japanese and English learners) tonal experience, seems to point to a universal challenge that L2 learners face in acquiring Mandarin Tone 3.

Chinese proficiency has no effect on perceptual accuracy, again echoing the findings in Wu et al. (2014) that the assimilation of Mandarin Tone 3 among Thai native listeners does not change as Mandarin experiences accumulate. Our finding is inconsistent with studies showing the learning path of L2 learners in tone perception (K. Chen & Yang, 2021; Wiener, 2017; Yu et al., 2019) and highlighted the fossilization aspect of speech learning in a second language, which is frequently documented in studies of second language speech learning (Rahal & Smaoui, 2020). Unlike response accuracy, reaction time indicated comparable performance between the two groups. This indicates that while Thai L2 learners could respond as quickly as native speakers, they cannot accurately distinguish tones, especially in challenging conditions such as loud speech. This incongruence in reaction time and accuracy reveals the learners’ exact difficulty with Mandarin tones: they do not hesitate more than native listeners but fail to identify the correct tones. In other words, Thai L2 learners consume no more energy (from the perspective of time) than native listeners but struggle to integrate rapid auditory processing with precise tonal distinctions. Our finding is in reverse to the existing literature, which showed lower accuracy but prolonged reaction time and more consumption of energy among L2 learners than native speakers (Ling & Grüter, 2020; Peirce, 2007). The possible reason is the different tasks and materials used in the present study. In the present study, Mandarin tones were presented with extended durations, allowing the tonal information to unfold gradually and giving listeners sufficient time to respond. Future studies should examine this by presenting tones with shorter durations.

### 4.2. Effects of Speech Mode on the Perception of Tones

The results of our study indicate that native listeners’ tone identification was unaffected by speech mode, whereas Thai L2 learners showed reduced accuracy when perceiving loud speech, particularly in identifying Tone 3. Specifically, the accuracy of Thai L2 learners in distinguishing Tone 3 declined from 76% in normal speech to 66% in loud speech, with the majority of Tone 3 misidentifications being classified as Tone 2.

Consistently, Shih and Lu (2015) did not find perceptual significance of the acoustic enhancement in loud speech among native listeners. There are two possible reasons for this. First, the native listeners have already reached the ceiling effect when perceiving tone in normal speech (Chang et al., 2017; Z. E. Peng & Wang, 2019; Zheng & Samuel, 2018). Second, the enhancement is not perceptually salient such that the acoustic modification in loud speech is not perceptually meaningful. Thus, we extend the theory of H&H by showing that it is not always true that hyper-articulation benefits perception. In cases where modifications are not critical for category discrimination or listeners have already reached the ceiling effect, the hyper-articulation, though observed in production, does not have perceptual significance. Future study is needed to clarify the specific reason.

Mandarin tones in loud speech even exacerbated confusion between Tone 2 and Tone 3 for Thai L2 learners, primarily due to the acoustic modifications it introduced. Specifically, Tone 3, typically characterized by a low pitch, began to exhibit features of Tone 2, such as elevated pitch height, in loud speech. This overlap in acoustic properties made it more challenging to differentiate between the two tones accurately. Our findings add more supportive data to the existing literature showing that L2 learners of Mandarin tend to use pitch height, instead of pitch contour, in discriminating tones (A. Li, 2025; Wang et al., 2006).

Our findings diverge from Shih and Lu (2015) and previous studies on FDS (S. Figueiredo, 2019; Y. Zhu & Mok, 2022), which have demonstrated that the expanded phonetic space improved perceptual accuracy among L2 learners. However, in our study, loud speech introduced more tonal confusion, particularly for Thai L2 learners, without changingthe performance of native listeners. This discrepancy between our study and studies on FDS may be attributed to fundamental differences in speech modes: while loud speech and Lombard speech seek to increase intelligibility by raising volume to overcome barriers such as noise or distance, FDS is closely linked to a didactic intention to aid foreigners in learning phonetic categories by expanding the space among them (M. Han et al., 2018; Tang et al., 2017). Unlike FDS, the main task of loud speech is to increase speech volume; thus, speakers might sacrifice the clarity of other acoustic dimensions to ensure that speech signals reach the listeners.

The inconsistency between Shih and Lu (2015) and the present study is probably caused by the manipulation of perceptual stimuli. In Shih and Lu (2015), the intensity of tones was not normalized. Consequently, the tones with larger TLD entailed stronger intensity than those with smaller TLD. The improvement in perceptual performance was thus attributed to the louder volume and/or other modifications as TLD increased. However, the perceptual influence of intensity and other acoustic modifications in loud speech could not be teased apart. It is likely that the tones with lower intensity in Shih and Lu (2015) were presented below the perceptual threshold for accurate identification by L2 learners. If so, the improvement in perceptual accuracy was likely due to increased intensity, rather than modifications in F0 or duration. In the present study, loud and normal tones were normalized concerning intensity. Therefore, we could tease out the confounding effect of intensity and ensure that all the tones were above the auditory threshold. When intensity was controlled, we observed that the F0 and duration modifications in loud tones caused more confusion between Tone 2 and Tone 3. Our study’s findings are more consistent with research on Lombard speech, which has similarly shown that increased speech volume can introduce acoustic distortions that hinder tone perception (Koenig & Fuchs, 2019).

For both native and L2 listener groups, the overall RT increased under loud speech conditions. However, this should not be interpreted as evidence that loud tones inherently require more time to process. Rather, the extended RT is likely attributable to increased vowel duration in loud speech, as tones are carried on the vowel segment. In other words, it is not the tonal complexity per se that delays responses, but the temporal stretching of the speech signal. This doubt derives from observation of the present study and the prior studies showing that vowel duration tends to lengthen across various speech styles—such as Infant-Directed Speech, Lombard Speech, and distance-directed speech (Shih & Lu, 2015; Tang et al., 2017)—which aligns with our findings for loud speech.

After controlling vowel duration, delayed reaction times remained only for Tone 3 and Tone 4 under loud speech conditions. This suggests that the processing of these tones requires more cognitive effort in loud speech, likely due to their increased acoustic variability. Interestingly, this effect was observed in both native listeners and L2 learners. It is possible that native listeners also experience momentary hesitation when exposed to tonal realizations that deviate from their expectations in normal speech, though this hesitation does not compromise their final identification accuracy. That is, while loud speech did not reduce native listeners’ tone recognition accuracy, its impact is nonetheless reflected in longer processing times. To our knowledge, no previous studies have specifically examined how speech mode influences listeners’ reaction times in tone perception. Our findings thus provide novel insights into the temporal dynamics of tone processing under altered speech conditions.

### 4.3. Implications of the Study

The present study has significant theoretical implications. First, we provide perceptual data to complement the assumptions in H & H theory. Although increased intensity, F0, and duration seem to be a universal enhancement in adverse communicative conditions, the enhancement of prosodic variation and tonal/vowel space depends on speech modality and speakers’ intention. Only the enhancement that is aimed at producing sharper contrast among speech categories results in expanded tonal/vowel space and perceptual improvement. Otherwise, the enhancement would not improve perceptual performance. Second, we offer insights into the theories of L2 speech learning. We emphasized the interrupting role of speech variability caused by speech modes in the perception of L2 speech. Besides, we propose that caution is needed when we infer the perceptual significance of the observed speech enhancement from speakers, especially for L2 learners. Among L2 learners, the cue weighting strategy is not the same as that with native listeners. Consequently, when multiple dimensions were concurrently adjusted in speech, it is very likely that L2 learners do not benefit from the intended adjustments, but are interrupted by the unintended adjustments.

The results of this study also have pedagogical implications for teaching practices aimed at Chinese L2 learners. Given that loud speech contributes to the confusion between Tone 2 and Tone 3, it is recommended that educators employ normal speech when emphasizing critical linguistic information. This approach is essential to ensure clarity in phonetic instruction, facilitating better tone perception among learners.

However, classrooms are especially noisy workplaces (Rantala et al., 2015; Shield & Dockrell, 2004). In educational contexts, loud speech is often seen as an effective tool for capturing students’ attention in noisy classrooms. Using ISO 9921 categories (International Organization for Standardization, 2018) for speech loudness, Kristiansen et al. (2014) estimated that teachers spoke with an equivalent voice level corresponding to a raised voice in 61% of the lessons (excluding Sports). While it can serve to engage learners and signal important information, it paradoxically hampers the accurate perception of phonetic details, especially for second language learners. This study has shown that loud speech can distort tonal distinctions, making it difficult for learners to identify subtle variations that are crucial for correct tone recognition. Therefore, educators must strike a balance between engaging teaching methods and the necessity for precise auditory input. Alternatively, integrating multimodal teaching strategies, such as the use of gestures or visual aids, can enhance understanding and retention of key tonal distinctions. Recent studies have documented the exploration of a range of multimodal strategies to enhance L2 learners’ acquisition of Mandarin tones. For instance, Baills and colleagues have demonstrated that pitch gestures—hand movements that iconically represent tonal contours—can significantly facilitate both tone identification and word recall in beginner learners of Mandarin (Baills et al., 2019). These findings suggest that non-verbal, perceptually salient cues such as gestures can enhance tone learning through multisensory integration mechanisms. Drawing learners’ attention to critical content through methods that do not hamper the perception of critical phonetic details supports L2 learners in developing more accurate perceptual skills.

### 4.4. Limitations

This study has three main limitations. First, although we successfully separated intensity from other acoustic features, our experimental design did not independently manipulate or isolate the remaining parameters (e.g., F0 range, mean F0, minimum and maximum F0, and F0 slope or curvature). Instead, loud speech was treated as a naturally co-occurring set of acoustic modifications. As a result, while our findings offer insight into the overall influence of loud speech on L2 tone identification, they do not permit causal conclusions about the specific contributions of individual acoustic features. Future research employing controlled resynthesis or parametric manipulation of distinct F0 dimensions will be necessary to clarify their respective effects and more precisely identify the mechanisms driving the observed perceptual outcomes. Second, the current study relied solely on end-point behavioral data, leaving the real-time processing dynamics of tone perception unexamined. To gain a deeper understanding of the cognitive processes involved, future work should incorporate online measures such as eye-tracking or event-related potentials (ERP). Third, since we do not have data directly showing the perception of loud vs. normal speech in the L1 of these Thai learners, we are unable to compare how loud speech modulates tone perception in their native language versus a second language. Future studies could address this gap by including L1 data, which would provide a more comprehensive understanding of the cross-linguistic effects of speech modulation on tone perception.

## 5. Conclusions

The present study shows that increasing the speaking voice cannot improve the ability of tone perception in Thai L2 learners. Instead, loud speech aggravates the confusion of Mandarin Tone 2 and Tone 3. The longer reaction time for all Mandarin tones in loud speech also explains that more cognitive resources are occupied in perceiving and processing louder tonal stimuli. Future study is needed to focus on the broader population (e.g., L2 learners from non-tonal languages) and explore more universal conclusions.

## Figures and Tables

**Table 1:** Outputs of post hoc comparisons.

Contrast	Estimate (*ß*)	SE	z-Value	*p*-Value
Loud Tone 1 Learner—Normal Tone 1 Learner	−0.678	0.184	−3.686	0.021 *
Loud Tone 2 Learner—Normal Tone 2 Learner	0.137	0.105	1.308	0.996
Loud Tone 3 Learner—Normal Tone 3 Learner	0.548	0.080	−6.887	<0.001 ***
Loud Tone 4 Learner—Normal Tone 4 Learner	0.411	0.164	2.505	0.468
Loud Tone 1 Native—Normal Tone 1 Native	−0.034	0.190	−0.177	1.000
Loud Tone 2 Native—Normal Tone 2 Native	0.413	0.147	2.815	0.260
Loud Tone 3 Native—Normal Tone 3 Native	−0.279	0.131	−2.121	0.752
Loud Tone 4 Native—Normal Tone 4 Native	0.345	0.215	1.604	0.968
Loud Tone 1 Learner—Loud Tone 1 Native	−0.598	0.283	−2.114	0.756
Normal Tone 1 Learner—Normal Tone 1 Native	0.047	0.305	0.155	1.000
Loud Tone 2 Learner—Loud Tone 2 Native	−1.089	0.258	−4.222	0.003 **
Normal Tone 2 Learner—Normal Tone 2 Native	−0.813	0.252	−3.227	0.091
Loud Tone 3 Learner—Loud Tone 3 Native	−1.843	0.233	−7.915	<0.001 ***
Normal Tone 3 Learner—Normal Tone 3 Native	−1.573	0.241	−6.534	<0.001 ***
Loud Tone 4 Learner—Loud Tone 4 Native	−0.647	0.296	−2.185	0.708
Normal Tone 4 Learner—Normal Tone 4 Native	−0.713	0.275	−2.592	0.404

* *p* < 0.05, ** *p* < 0.01, *** *p* < 0.001. Formula: pairwise~Mode × Tone × Group.

**Table 2:** Contrasts in Estimated Marginal Means of Interaction Effects of Variables for RT.

Contrast	Estimate(ß)	SE	z-Value	*p*-Value
Loud Tone 1 Learner—Normal Tone 1 Learner	91.54	16.4	5.589	<0.001 ***
Loud Tone 2 Learner—Normal Tone 2 Learner	59.37	17.1	3.463	0.044 *
Loud Tone 3 Learner—Normal Tone 3 Learner	101.92	18.1	5.634	<0.001 ***
Loud Tone 4 Learner—Normal Tone 4 Learner	137.80	16.5	8.344	<0.001 ***
Loud Tone 1 Native—Normal Tone 1 Native	93.99	16.3	5.773	<0.001 ***
Loud Tone 2 Native—Normal Tone 2 Native	75.90	16.4	4.622	<0.001 ***
Loud Tone 3 Native—Normal Tone 3 Native	139.65	16.5	8.466	<0.001 ***
Loud Tone 4 Native—Normal Tone 4 Native	148.57	16.3	9.123	<0.001 ***
Loud Tone 1 Learner—Loud Tone 1 Native	56.07	49.8	1.125	0.999
Normal Tone 1 Learner—Normal Tone 1 Native	58.52	50.1	1.168	0.999
Loud Tone 2 Learner—Loud Tone 2 Native	162.84	50.1	3.248	0.086
Normal Tone 2 Learner—Normal Tone 2 Native	179.36	50.3	3.562	0.032 *
Loud Tone 3 Learner—Loud Tone 3 Native	125.24	50.5	2.479	0.487
Normal Tone 3 Learner—Normal Tone 3 Native	162.97	50.6	3.223	0.093
Loud Tone 4 Learner—Loud Tone 4 Native	57.31	50.0	1.147	0.999
Normal Tone 4 Learner—Normal Tone 4 Native	68.08	50.2	1.356	0.994

* *p* < 0.05, ** *p* < 0.01, *** *p* < 0.001. Formula: pairwise~Mode × Tone × Group.

**Table 3:** Contrasts in Estimated Marginal Means of Interaction Effects of Variables for the difference between RT and tonal duration.

Contrast	Estimate(ß)	SE	z-Value	*p*-Value
Loud Tone 1 Learner—Normal Tone 1 Learner	0.036	0.010	3.750	0.017 *
Loud Tone 2 Learner—Normal Tone 2 Learner	0.014	0.011	1.318	0.995
Loud Tone 3 Learner—Normal Tone 3 Learner	0.046	0.013	3.650	0.024 *
Loud Tone 4 Learner—Normal Tone 4 Learner	0.052	0.010	5.223	<0.001 ***
Loud Tone 1 Native—Normal Tone 1 Native	0.030	0.009	3.227	0.091
Loud Tone 2 Native—Normal Tone 2 Native	0.025	0.010	2.567	0.422
Loud Tone 3 Native—Normal Tone 3 Native	0.081	0.010	8.338	<0.001 ***
Loud Tone 4 Native—Normal Tone 4 Native	0.057	0.009	5.982	<0.001 ***

* *p* < 0.05, ** *p* < 0.01, *** *p* < 0.001. Formula: pairwise~Mode × Tone × Group.

## Data Availability

According to the Non-Disclosure Agreement (NDA), the data cannot be disclosed through public channels. The corresponding author can provide the datasets used and/or analyzed during the current study upon reasonable request.

## Notes

1 Cantonese has six distinct tones in open syllables (syllables ending in a vowel or nasal), including high level, high rising, mid level, low falling, low rising, and low level (Zhao & Jurafsky, 2009).

2 Here, the term curvature refers to a nonlinear feature of a tone contour. For example, Tone 3 falls down and then rises towards the end, which is also called dipping. The difference in Tone 2 and Tone 3 in curvature lies in the sharpness of the valley of the falling-rising contour.

3 Given that the dataset comprises recordings from 20 individual speakers, we acknowledge the potential variability this introduces in terms of pitch range, voice quality, articulation habits, and prosodic patterns. To account for such speaker-related variation, we implemented several control measures during data collection and analysis. First, only speakers with clear tone production (as pre-screened by native Mandarin listeners) were selected to ensure tonal fidelity. Second, in the perceptual analysis, mixed-effects modeling was employed with the speaker included as a random effect, allowing us to statistically account for individual differences across talkers. This approach enabled us to isolate the effects of speech type (loud vs. plain) and listener group (L1 vs. L2) while controlling for inter-speaker variability. Therefore, while natural variation among speakers is inevitable, our methodological design aimed to minimize its confounding influence on the results.

## Supplementary Materials

The following supporting information can be downloaded at: https://www.mdpi.com/article/10.3390/bs15081062/s1, Table S1. ANOVA Results of glmer model for Accuracy by Tone, Mode, and Group. Table S2. ANOVA Results of lmer model for Reaction time by Tone, Mode, and Group. Table S3. ANOVA Results of glmer model for Accuracy of L2 learners by Tone, Mode, Hsk level, and Gender. Table S4. ANOVA Results of lmer model for Reaction time of L2 learners by Tone, Mode, Hsk level, and Gender.

## References

[B1-behavsci-15-01062] Baills F., Suarez-Gonzalez N., Gonzalez-Fuente S., Prieto P. (2019). Observing and producing pitch gestures facilitates the learning of Mandarin Chinese tones and words. Studies in Second Language Acquisition.

[B2-behavsci-15-01062] Belz M., Ebert M., Müller M., Sun J., Terada M., Xia Q. (2023). Reduced vowel space in video conferences via Zoom: Evidence from read speech. JASA Express Letters.

[B3-behavsci-15-01062] Blicher D. L., Diehl R. L., Cohen L. B. (1990). Effects of syllable duration on the perception of the Mandarin Tone 2/Tone 3 distinction: Evidence of auditory enhancement. Journal of Phonetics.

[B4-behavsci-15-01062] Cao M., Pavlik P. I., Bidelman G. M. (2024). Enhancing lexical tone learning for second language speakers: Effects of acoustic properties in Mandarin tone perception. Frontiers in Psychology.

[B5-behavsci-15-01062] Carding P., Mathieson L. (2018). Voice and speech production. Scott-Brown’s otorhinolaryngology and head and neck surgery.

[B6-behavsci-15-01062] Chang Y. H. S., Yao Y., Huang B. H. (2017). Effects of linguistic experience on the perception of high-variability non-native tones. The Journal of the Acoustical Society of America.

[B7-behavsci-15-01062] Chao Y. R. (1965). A grammar of spoken Chinese.

[B8-behavsci-15-01062] Chen F., Peng G. (2016). Context effect in the categorical perception of Mandarin tones. Journal of Signal Processing Systems.

[B9-behavsci-15-01062] Chen J., Best C. T., Antoniou M. (2023). Phonological and phonetic contributions to Thai-naïve Mandarin and Vietnamese speakers’ imitation of Thai lexical tones: Effects of memory load and stimulus variability. Laboratory Phonology.

[B10-behavsci-15-01062] Chen K., Yang C. (2021). The effect of fundamental frequency on Mandarin intelligibility by L2 learners in quiet and noise environments: A pilot study. The acquisition of Chinese as a second language pronunciation: Segments and prosody.

[B11-behavsci-15-01062] Chen X., Liu Y., Zhu S. (2020). The effect of noise conditions on lexical tone perception of Mandarin L2 learners of Indonesian.

[B12-behavsci-15-01062] Chow R. W. C., Liu Y., Ning J. H. (2019). The categorical perception of mandarin tone 2 and tone 3 by tonal and non-tonal listeners. Proceedings of the 19th ICPhS.

[B13-behavsci-15-01062] Cox C., Bergmann C., Fowler E., Keren-Portnoy T., Roepstorff A., Bryant G., Fusaroli R. (2023). A systematic review and Bayesian meta-analysis of the acoustic features of infant-directed speech. Nature Human Behaviour.

[B14-behavsci-15-01062] Dromey C., Ramig L. O. (1998). Intentional changes in sound pressure level and rate: Their impact on measures of respiration, phonation, and articulation. Journal of Speech.

[B15-behavsci-15-01062] Dromey C., Ramig L. O., Johnson A. B. (1995). Phonatory and Articulatory changes associated with increased vocal intensity in Parkinson disease: A case study. Journal of Speech, Language, and Hearing Research.

[B16-behavsci-15-01062] Fant G. (1971). Notes on the Swedish vowel system. Form and substance: Phonetic and linguistic papers.

[B17-behavsci-15-01062] Figueiredo S. (2019). Competition strategies during writing in a second language: Age and levels of complexity. Languages.

[B18-behavsci-15-01062] Figueiredo S. (2024). Topic modelling and sentiment analysis during COVID-19 revealed emotions changes for public health. Scientific Reports.

[B19-behavsci-15-01062] Figueiredo S. A. D. B., Silva C. F. D. (2009). Cognitive differences in second language learners and the critical period effects. L1-Educational Studies in Language and Literature.

[B20-behavsci-15-01062] Gandour J. (1983). Tone perception in far Eastern languages. Journal of Phonetics.

[B21-behavsci-15-01062] Garnier M., Ménard L., Alexandre B. (2018). Hyper-articulation in Lombard speech: An active communicative strategy to enhance visible speech cues?. The Journal of the Acoustical Society of America.

[B22-behavsci-15-01062] Gottfried T. L., Suiter T. L. (1997). Effect of linguistic experience on the identification of Mandarin Chinese vowels and tones. Journal of Phonetics.

[B23-behavsci-15-01062] Hallé P. A., Chang Y.-C., Best C. T. (2004). Identification and discrimination of Mandarin Chinese tones by Mandarin Chinese vs. French listeners. Journal of Phonetics.

[B25-behavsci-15-01062] Han J. I., Tsukada K. (2020). Lexical representation of Mandarin tones by non-tonal second-language learners. The Journal of the Acoustical Society of America.

[B24-behavsci-15-01062] Han M., De Jong N. H., Kager R. (2018). Lexical tones in Mandarin Chinese infant-directed speech: Age-related changes in the second year of life. Frontiers in Psychology.

[B26-behavsci-15-01062] Hao Y. C. (2012). Second language acquisition of Mandarin Chinese tones by tonal and non-tonal language speakers. Journal of Phonetics.

[B27-behavsci-15-01062] Hao Y. C. (2018). Contextual effect in second language perception and production of Mandarin tones. Speech Communication.

[B28-behavsci-15-01062] Howie J. M. (1976). Acoustical studies of Mandarin vowels and tones.

[B29-behavsci-15-01062] Huang J., Holt L. L. (2009). General perceptual contributions to lexical tone normalization. The Journal of the Acoustical Society of America.

[B30-behavsci-15-01062] Huber J. E., Chandrasekaran B. (2006). Effects of increasing sound pressure level on lip and jaw movement parameters and consistency in young adults. Journal of Speech, Language, and Hearing Research.

[B31-behavsci-15-01062] International Organization for Standardization (2018). Occupational health and safety management systems—Requirements with guidance for use.

[B32-behavsci-15-01062] Jiang S. (2023). Cue weighting in Mandarin tone perception: A comparison between native speakers and learners of Mandarin. 20th International Congress of Phonetic Sciences.

[B33-behavsci-15-01062] Koenig L. L., Fuchs S. (2019). Vowel formants in normal and loud speech. Journal of Speech, Language, and Hearing Research.

[B34-behavsci-15-01062] Kristiansen J., Lund S. P., Persson R., Shibuya H., Nielsen P. M., Scholz M. (2014). A study of classroom acoustics and school teachers’ noise exposure, voice load and speaking time during teaching, and the effects on vocal and mental fatigue development. International Archives of Occupational and Environmental Health.

[B35-behavsci-15-01062] Ladefoged P., McKinney N. P. (1963). Loudness, sound pressure, and subglottal pressure in speech. The Journal of the Acoustical Society of America.

[B36-behavsci-15-01062] Lee C.-Y., Tao L., Bond Z. S. (2009). Speaker variability and context in the identification of fragmented Mandarin tones by native and non-native listeners. Journal of Phonetics.

[B37-behavsci-15-01062] Leung K. K., Wang Y. (2020). Production-perception relationship of Mandarin tones as revealed by critical perceptual cues. The Journal of the Acoustical Society of America.

[B38-behavsci-15-01062] Li A. (2025). Is pitch height or pitch contour a challenge? Production of Mandarin tones in Hani–Mandarin bilingual children. Journal of Speech, Language, and Hearing Research.

[B39-behavsci-15-01062] Li B., Zhang C. (2010). Effects of F0 dimensions in perception of Mandarin tones. 2010 7th International Symposium on Chinese Spoken Language Processing.

[B40-behavsci-15-01062] Li M., Wang W., Tao S., Dong Q., Guan J., Liu C. (2016). Mandarin Chinese vowel-plus-tone identification in noise: Effects of language experience. Hearing Research.

[B41-behavsci-15-01062] Li X., To C. K. S., Ng M. L. (2017). Effects of L1 tone on perception of L2 tone-a study of Mandarin tone learning by native Cantonese children. Bilingualism: Language and Cognition.

[B42-behavsci-15-01062] Lindblom B. (1983). Economy of speech gestures. The production of speech.

[B43-behavsci-15-01062] Lindblom B. (1990). Explaining phonetic variation: A sketch of the H&H theory. Speech production and speech modelling.

[B44-behavsci-15-01062] Ling W., Grüter T. (2019). Learning words with lexical tone: Is manipulation of attentional focus beneficial?. Proceedings of the 44th Boston University Conference on Language Development.

[B45-behavsci-15-01062] Liu S., Samuel A. G. (2004). Perception of Mandarin Lexical Tones when F0 Information is Neutralized. Language and Speech.

[B46-behavsci-15-01062] Lunichkin A. M., Gvozdeva A. P., Andreeva I. G. (2023). The impact of visual estimates of talker-to-listener distance on fundamental frequency in noise. Human Physiology.

[B47-behavsci-15-01062] Mao Y., Xu L. (2017). Lexical tone recognition in noise in normal-hearing children and prelingually deafened children with cochlear implants. International Journal of Audiology.

[B48-behavsci-15-01062] Marklund E., Gustavsson L. (2020). The dynamics of vowel hypo- and hyperarticulation in Swedish infant-directed speech to 12-month-olds. Frontiers in Communication.

[B49-behavsci-15-01062] Mefferd A. S. (2017). Tongue- and jaw-specific contributions to acoustic vowel contrast changes in the diphthong /ai/ in response to slow, loud, and clear speech. Journal of Speech, Language, and Hearing Research.

[B50-behavsci-15-01062] Mota A. F. D. B., Pellicani A. D., Dornelas R., Ricz L. N. A. (2021). Vocal teacher production condition in differents functional situations. CoDAS.

[B51-behavsci-15-01062] Myers B. R., Finnegan E. M. (2015). The effects of articulation on the perceived loudness of the projected voice. Journal of Voice.

[B52-behavsci-15-01062] Neel A. T. (2009). Effects of loud and amplified speech on sentence and word intelligibility in Parkinson disease. Journal of Speech, Language, and Hearing Research.

[B53-behavsci-15-01062] Oxenham A. J. (2012). Pitch perception. The Journal of Neuroscience.

[B54-behavsci-15-01062] Pagel L., Roessig S., Mücke D. (2024). The encoding of prominence relations in supra-laryngeal articulation across speaking styles. Laboratory Phonology.

[B55-behavsci-15-01062] Peirce J. W. (2007). PsychoPy—Psychophysics software in Python. Journal of Neuroscience Methods.

[B56-behavsci-15-01062] Pelzl E., Lau E. F., Guo T., DeKeyser R. (2021). Even in the best-case scenario L2 learners have persistent difficulty perceiving and utilizing tones in Mandarin: Findings from behavioral and event-related potentials experiments. Studies in Second Language Acquisition.

[B57-behavsci-15-01062] Peng S. C., Tomblin J. B., Cheung H., Lin Y. S., Wang L. S. (2004). Perception and production of Mandarin tones in prelingually deaf children with cochlear implants. Ear and Hearing.

[B58-behavsci-15-01062] Peng Z. E., Wang L. M. (2019). Listening effort by native and nonnative listeners due to noise, reverberation, and talker foreign accent during English speech perception. Journal of Speech, Language, and Hearing Research.

[B59-behavsci-15-01062] Piazza G., Martin C. D., Kalashnikova M. (2022). The acoustic features and didactic function of foreigner-directed speech: A scoping review. Journal of Speech, Language, and Hearing Research.

[B60-behavsci-15-01062] Picheny M. A., Durlach N. I., Braida L. D. (1986). Speaking clearly for the hard of hearing II: Acoustic characteristics of clear and conversational speech. Journal of Speech, Language, and Hearing Research.

[B61-behavsci-15-01062] Rahal A., Smaoui C. (2020). Assessing the role of selective fossilization hypothesis in determining fossilizable phonetic errors in Tunisian EFL learners’ oral output. Language Testing.

[B62-behavsci-15-01062] Rantala L. M., Hakala S., Holmqvist S., Sala E. (2015). Classroom noise and teachers’ voice production. Journal of Speech, Language, and Hearing Research.

[B63-behavsci-15-01062] Schulman R. (1989). Articulatory dynamics of loud and normal speech. The Journal of the Acoustical Society of America.

[B64-behavsci-15-01062] Shen C., Cooke M., Janse E. (2023). Speaking in the presence of noise: Consistency of acoustic properties in clear-Lombard speech over time. The Journal of the Acoustical Society of America.

[B65-behavsci-15-01062] Shen G., Froud K. (2016). Categorical perception of lexical tones by English learners of Mandarin Chinese. The Journal of the Acoustical Society of America.

[B66-behavsci-15-01062] Shen G., Froud K. (2019). Electrophysiological correlates of categorical perception of lexical tones by English learners of Mandarin Chinese: An ERP study. Bilingualism: Language and Cognition.

[B67-behavsci-15-01062] Shen X. S., Lin M. (1991). A perceptual study of Mandarin Tones 2 and 3. Language and Speech.

[B68-behavsci-15-01062] Shen X. S., Lin M., Yan J. (1993). *F* 0 turning point as an *F* 0 cue to tonal contrast: A case study of Mandarin tones 2 and 3. The Journal of the Acoustical Society of America.

[B70-behavsci-15-01062] Shi F. (2019). Auditory patterns: A preliminary study on the characteristics of Chinese speech perception.

[B69-behavsci-15-01062] Shield B., Dockrell J. E. (2004). External and internal noise surveys of London primary schools. The Journal of the Acoustical Society of America.

[B71-behavsci-15-01062] Shih C., Lu H.-Y. D. (2015). Effects of talker-to-listener distance on tone. Journal of Phonetics.

[B72-behavsci-15-01062] Smith D., Burnham D. (2012). Faciliation of Mandarin tone perception by visual speech in clear and degraded audio: Implications for cochlear implants. The Journal of the Acoustical Society of America.

[B73-behavsci-15-01062] So C. K., Best C. T. (2010). Cross-language perception of non-native tonal contrasts: Effects of native phonological and phonetic influences. Language and Speech.

[B74-behavsci-15-01062] Sun Y., Kyaw W. T., Zhang J., Sagisaka Y. (2018). Analysis of L2 learners’ progress of distinguishing Mandarin Tone 2 and Tone 3. 19th Annual Conference of the International Speech Communication, INTERSPEECH 2018.

[B75-behavsci-15-01062] Tang P., Xu Rattanasone N., Yuen I., Demuth K. (2017). Acoustic realization of Mandarin neutral tone and tone sandhi in infant-directed speech and Lombard speech. The Journal of the Acoustical Society of America.

[B76-behavsci-15-01062] Ternström S., Bohman M., Södersten M. (2006). Loud speech over noise: Some spectral attributes, with gender differences. The Journal of the Acoustical Society of America.

[B77-behavsci-15-01062] Tong X., McBride C., Burnham D. (2014). Cues for lexical tone perception in children: Acoustic correlates and phonetic context effects. Journal of Speech, Language, and Hearing Research.

[B78-behavsci-15-01062] Tsao F.-M. (2017). Perceptual improvement of lexical tones in infants: Effects of tone language experience. Frontiers in Psychology.

[B79-behavsci-15-01062] Tupper P., Leung K., Wang Y., Jongman A., Sereno J. A. (2018). Identifying the distinctive acoustic cues of Mandarin tones. The Journal of the Acoustical Society of America.

[B80-behavsci-15-01062] Tupper P., Leung K., Wang Y., Jongman A., Sereno J. A. (2020). Characterizing the distinctive acoustic cues of Mandarin tones. The Journal of the Acoustical Society of America.

[B81-behavsci-15-01062] Uchihara T., Karas M., Thomson R. I. (2024). Does perceptual high variability phonetic training improve L2 speech production? A meta-analysis of perception-production connection. Applied Psycholinguistics.

[B82-behavsci-15-01062] Uther M., Knoll M. A., Burnham D. (2007). Do you speak E-NG-L-I-SH? A comparison of foreigner- and infant-directed speech. Speech Communication.

[B83-behavsci-15-01062] Van Engen K. J., Bradlow A. R. (2007). Sentence recognition in native- and foreign-language multi-talker background noise. The Journal of the Acoustical Society of America.

[B84-behavsci-15-01062] Wang Y., Sereno J. A., Jongman A. (2006). L2 acquisition and processing of Mandarin tones. Handbook of Chinese psycholinguistics.

[B85-behavsci-15-01062] Whalen D. H., Xu Y. (1992). Information for Mandarin tones in the amplitude contour and in brief segments. Phonetica.

[B86-behavsci-15-01062] Wiener S. (2017). Changes in early L2 cue-weighting of non-native speech: Evidence from learners of Mandarin Chinese. 18th Annual Conference of the International Speech Communication Association (INTERSPEECH 2017).

[B87-behavsci-15-01062] Wilson Black J., Hay J., Clark L., Brand J. (2024). The overlooked effect of amplitude on within-speaker vowel variation. Linguistics Vanguard.

[B88-behavsci-15-01062] Wu X., Lin H. (2008). Perception of Mandarin tones by Mandarin and English listeners. Journal of Chinese Language and Computing.

[B89-behavsci-15-01062] Wu X., Munro M. J., Wang Y. (2014). Tone assimilation by Mandarin and Thai listeners with and without L2 experience. Journal of Phonetics.

[B90-behavsci-15-01062] Xu Y. (1997). Contextual tonal variations in Mandarin. Journal of Phonetics.

[B91-behavsci-15-01062] Yu K., Li L., Chen Y., Zhou Y., Wang R., Zhang Y., Li P. (2019). Effects of native language experience on Mandarin lexical tone processing in proficient second language learners. Psychophysiology.

[B92-behavsci-15-01062] Zhang H., Liu W. T., Shi J. J., Ye Y. H. (2023). F0 change of lexical tones in loud speech *[Conference presentation]*. The Second International Conference on Tone and Intonation.

[B93-behavsci-15-01062] Zhang H., Wiener S., Holt L. L. (2022). Adjustment of cue weighting in speech by speakers and listeners: Evidence from amplitude and duration modifications of Mandarin Chinese tone. Journal of the Acoustical Society of America.

[B94-behavsci-15-01062] Zhao Y., Jurafsky D. (2009). The effect of lexical frequency and Lombard reflex on tone hyperarticulation. Journal of Phonetics.

[B95-behavsci-15-01062] Zheng Y., Samuel A. G. (2018). The effects of ethnicity, musicianship, and tone language experience on pitch perception. Quarterly Journal of Experimental Psychology.

[B96-behavsci-15-01062] Zhu S., Wong L. L., Chen F. (2014). Tone identification in Mandarin-speaking children with profound hearing impairment. International Journal of Pediatric Otorhinolaryngology.

[B97-behavsci-15-01062] Zhu Y., Mok P. (2022). The role of prosody across languages. The Routledge handbook of second language acquisition and speaking.

