# Supplementary material for: Identification of Mandarin Tones in Loud Speech for Native Speakers and Second Language Learners
Source: Behav Sci (Basel). 2025 Aug 5;15(8):1062. doi: 10.3390/bs15081062 (PMC12383229; doi:10.3390/bs15081062)
Supplement: Supplementary file 1 [file behavsci-15-01062-s001.zip › behavsci-3660608-supplementary.pdf]

Table S1. ANOVA Results of glmer model for Accuracy by Tone, Mode, and Group.

Measure	df_1	df_2	F	P
Tone	3	35636	42.63	< .001***
Mode	1	35636	10.88	< .001***
Group	1	35636	33.05	< .001***
Tone × Mode	3	35636	23.23	< .001***
Tone × Group	3	35636	25.89	< .001***
Mode × Group	1	35636	8.40	.004**
Tone × Mode × Group	3	35636	1.26	.288

*$p < .05$. **$p < .01$. ***$p < .001$.

Response accuracy ~ Group * Tone * Mode + (1 + Group + Tone + Mode | Subject)

Table S2. ANOVA Results of lmer model for Reaction time by Tone, Mode, and Group.

Measure	df_1	df_2	F	P
Tone	3	25.5	22.39	< .001***
Mode	1	26.6	57.26	< .001***
Group	1	14.5	4.89	.044*
Tone × Mode	3	26376.5	25.40	< .001***
Tone × Group	3	8316.2	50.70	< .001***
Mode × Group	1	7662.5	4.41	.036*
Tone × Mode × Group	3	26377.9	1.17	.318

*$p < .05$. **$p < .01$. ***$p < .001$.

Reaction time ~ Group * Tone * Mode + (1 + Group + Tone + Mode | Subject)

Table S3. ANOVA Results of glmer model for Accuracy of L2 learners by Tone, Mode, Hsk level, and Gender.

Measure	df_1	df_2	F	P
Tone	3	18199	50.07	< .001***
Mode	1	18199	20.91	< .001***
HSK_level	1	18199	0.69	.406
Gender	1	18199	0.09	.767
Tone × Mode	3	18199	18.00	< .001***
Mode × HSK_level	1	18199	5.70	.020*
Tone × Mode × Group	3	18199	4.26	.005*

*$p < .05$. **$p < .01$. ***$p < .001$.

Response accuracy ~ Tone * Mode*HSKlevel + Gender + Gender:Tone + Gender:Mode + Gender:Tone : Mode +(1 + Tone + Mode | Subject)

Table S4. ANOVA Results of lmer model for Reaction time of L2 learners by Tone, Mode, Hsk level, and Gender.

Measure	df_1	df_2	*F*	*P*
Tone	3	18.8	0.47	.704
Mode	1	19.3	3.72	.068
HSK_level	1	19.0	0.01	.943
Gender	1	19.0	0.65	.431
Tone × Mode	3	18119.8	3.10	.026*
Tone × HSK level	3	18.9	1.20	.336
Mode × HSK_level	1	19.4	1.07	.314
Tone × Gender	3	18.7	0.14	.935
Mode × Gender	1	19.0	3.85	.064
Tone × Mode × HSK level	3	18119.8	3.57	.013*
Tone × Mode × Gender	3	18119.8	3.39	.017*

$^{*}p < .05$. $^{**}p < .01$. $^{***}p < .001$.

Reaction time ~ Tone * Mode*HSKlevel + Gender + Gender:Tone + Gender:Mode + Gender:Tone : Mode +(1 + Tone + Mode | Subject)
